# Evaluation of Prognostic Factors for Clinical Pregnancy Rate Following Artificial Insemination by Husband in the Chinese Population

**DOI:** 10.3389/fmed.2021.638560

**Published:** 2021-05-10

**Authors:** Yumei Luo, Shunhong Wu, Jingru Yuan, Hua Zhou, Yufang Zhong, Mimi Zhang, Qing Li, Xia Xu, Xiaofang Sun, Detu Zhu

**Affiliations:** ^1^Department of Obstetrics and Gynecology, Key Laboratory for Major Obstetric Diseases of Guangdong Province, The Third Affiliated Hospital of Guangzhou Medical University, Guangzhou, China; ^2^Key Laboratory of Reproduction and Genetics of Guangdong Higher Education Institutes, The Third Affiliated Hospital of Guangzhou Medical University, Guangzhou, China; ^3^Kingmed School of Laboratory Medicine, Guangzhou Medical University, Guangzhou, China

**Keywords:** assisted reproduction, intrauterine insemination, artificial insemination by husband, pregnancy rate, semen analysis, logistic regression

## Abstract

**Background:** To determine the independent prognostic factors and develop a multivariate logistic regression model for predicting successful pregnancy following artificial insemination by husband (AIH) in infertile Chinese couples.

**Methods:** A total of 3,015 AIH cycles with superovulation from 1,853 infertile Chinese couples were retrospectively analyzed. The clinical characteristics and sperm parameters were compared between the pregnant and non-pregnant groups. Multivariate logistic regression analysis was performed to remove the confounding factors and create an equation to predict the successful pregnancy. Receiver operating characteristic (ROC) curves were constructed for evaluating the abilities for prognostic classification of the independent predictors and the equation.

**Results:** The overall pregnancy rate was 13.0%. The pregnancy rate of double intrauterine insemination (IUI) (18.9%) was significantly higher than that of single IUI (11.4%). The pregnancy rate of the stimulated cycle (14.4%) was significantly higher than that of the natural cycle (9.0%). The pregnancy rates of the age groups <40 years are ~3 times higher than that of the ≥40 years age group. Among sperm parameters, the influencing factors included straight-line velocity (VSL), sperm deformity index (SDI), and normal form rate (all *P* < 0.05). A multivariate logistic regression equation was created based on the above influencing factors. ROC analysis showed that the prognostic power of the equation is better than those of individual predictors.

**Conclusion:** Cycle treatment options, single/double IUI, female age, sperm VSL, SDI, and normal form rate could predict successful pregnancy following AIH in China. The multivariate logistic regression equation exhibited a greater value for prognostic classification than single predictors.

## Introduction

Due to its high cost-effectiveness, intrauterine artificial insemination (IUI) is currently the first-line treatment method of assisted reproduction and is often provided before *in vitro* fertilization (IVF) and intracytoplasmic sperm injection (ICSI) (1, 2). It can be classified into artificial insemination by husband (AIH) and artificial insemination by donor (AID) according to the source of the sperm. There are many factors influencing the clinical pregnancy rate following IUI, including age, infertility type, sperm quality, mature follicular number, endometrial thickness, and so on (3–7). However, the prognostic values of these factors remain controversial. For example, some studies have shown that double IUI and ovulation induction could increase the pregnancy rate of IUI (8, 9). On the contrary, some other studies argued that the single/double IUI and cycle treatment options did not influence it significantly (1, 10). On the other hand, abundant studies indicated that some semen parameters, such as semen volume, sperm motility, and morphology, could predict the pregnancy outcomes following IUI (8, 11–13); meanwhile, the other studies showed no significant prognostic value of these factors (14–16). In addition, some studies found that the combination of several influencing factors had the appreciable ability of prognostic classification (17). Therefore, it is also desired to create a multivariate mathematical model for predicting successful pregnancy following IUI.

In this study, we attempted to determine the independent predictors and create a multivariate logistic regression equation to predict the pregnancy rate following AIH in the Chinese population. A total of 1,853 infertile couples with 3,015 AIH treatment cycles between September 2018 and December 2019 were retrospectively evaluated. The multivariate logistic regression analysis showed that the influencing factors included cycle treatment options, single/double IUI, female age, sperm straight-line velocity (VSL), sperm deformity index (SDI), and normal form rate. Furthermore, the multivariate logistic regression equation exhibited a greater prognostic power than individual factors for predicting successful pregnancy following AIH.

## Materials and Methods

### Study Population

This was a retrospective cohort study enrolling infertile Chinese couples that underwent AIH treatment at the fertility clinic of the Third Affiliated Hospital of Guangzhou Medical University from September 2018 to December 2019. Prior to the enrollment, the patients were diagnosed with the cause of infertility, with investigations conducted as necessary to elicit etiology. Female patients had tubal patency confirmed by hysterosalpingogram, and men had a semen analysis. Causes of infertility were grouped into male factor, female factor (ovulatory dysfunction, endometriosis, cervical, and endometriosis after pelvic plastic surgery), combined male and female factors, and unexplained infertility.

All pregnancies were confirmed by positive beta-human chorionic gonadotropin (β-hCG) in the serum 14 days after AIH. Five weeks after the AIH, the ultrasound was reviewed. Demographic data such as the age, duration of infertility, semen routine parameters, and the IUI outcomes were extracted from the patients' records. Exclusion criteria were: (1) female partner had ovarian cysts detected by ultrasound examination; (2) uterine lesions such as submucosal leiomyoma; (3) a history of moderate to severe pelvic endometriosis. Records with incomplete or missing data were also excluded. Finally, a total of 3,015 AIH treatment cycles from 1,853 couples were analyzed.

### Semen Collection, Treatment, and Analysis

Semen specimens were harvested with masturbation in a collection room at the fertility clinic. The semen specimens were kept at 37°C temperature and were examined within half an hour of collection. After complete liquefaction, all samples were evaluated in a blinded fashion by a qualified technician to prevent the interobserver variation based on WHO 2010 criteria. 10 μL of semen was analyzed by computer-assisted sperm analysis (CASA; Hamilton Thorne HTCasa II 1.10.3). The sperm concentration, viability, morphology, and motility parameters, including total progressive motile sperm count (TPMSC), curvilinear velocity (VCL), straight-line velocity (VSL), average path velocity (VAP), linearity (LIN), straightness (STR), beat cross frequency (BCF), amplitude of lateral head displacement (ALH), sperm head area, sperm deformity index (SDI), teratozoospermia index (TZI), headpiece deformity rate (H), middle piece deformity rate (M), principal piece deformity rate (P), cytoplasm deformity rate (C), normal form rate, and survival rate were recorded. Semen specimens were treated by density gradient centrifugation.

### Superovulation and Intrauterine Insemination

Superovulation was performed with either hCG + Clomifene Citrate (CC), hCG + CC + human menopausal gonadotropin (hMG) or hCG + FSH/hMG. When more than one dominant follicle reached a diameter of 18 mm, or more than two dominant follicles reached a diameter of 17 mm, 6,000–10,000 IU of hCG was injected intramuscularly, and after 24–36 h, IUI was conducted.

After the bladder was emptied, the patient lied down in the bladder lithotomy position, the vulva was washed with normal saline, and the vagina, cervix, and fornix were wiped with a large cotton swab. A 1 ml syringe and an artificial insemination tube were connected in the uterine cavity. Aspirate 0.5 ml of the husband's sperm suspension. The catheter containing the suspension is slowly placed in the uterine cavity through the cervix and about 1 cm above the uterine cavity. After the semen is slowly injected into the uterus for 3–5 s, it is slowly withdrawn from the artificial insemination tube and speculum in the uterine cavity, and the patient is kept in the position of lowering the head and hips for about 30 min, and then can leave the operating room.

### Statistical Analysis

The SPSS v22.0 was used for statistical analysis. The results are presented as mean ± standard deviation (SD). The influence of categorical variables (such as clinical characteristics) were evaluated using the chi-square and Fisher's exact-test. The data distribution of continuous variables were assessed by Kolmogorov-Smirnov-test. The differences of abnormal distribution variables (such as sperm parameters) between the pregnant and non-pregnant groups were examined using the Mann-Whitney-test. Both categorical and continuous variables that might influence AIH pregnancy outcome were analyzed by backward stepwise multivariate binary logistic regression to remove confounding factors. Receiver operating characteristic (ROC) analysis was performed to evaluate the clinically acceptable cut-off value, sensitivity and specificity of each valuable variable. Statistical significance was accepted as *P* < 0.05.

## Results

### Association Between Clinical Characteristics and AIH Pregnancy Rate

A total of 3,015 AIH cycles were analyzed. The overall successful pregnancy rate was estimated to be 13.0% (392/3,015). Chi-square analysis was performed to evaluate the association between clinical characteristics and the pregnancy rate following AIH (Table 1). The results showed that the pregnancy rate of stimulated cycles was significantly higher than that of natural cycles (14.4 vs. 9.0%, *P* < 0.001); and the pregnancy rate of double IUI was significantly higher than that of single IUI (18.9 vs. 11.4%, *P* < 0.001). Meanwhile, the number of IUI cycles and the type of infertility displayed no significant impact on the pregnancy rate following AIH.

**Table 1:** Association between clinical characteristics and AIH pregnancy rate.

	**Total cycle**	**Pregnant cycle**	**χ^**2**^**	***p*-value**
**Cycle treatment options**				
Natural cycle	799	72 (9.3%)	15.303	**<0.001**
Stimulated cycle	2,216	320 (14.4%)		
**The number of IUI cycle**				
1	1,667	216 (13.0%)	1.188	0.756
2	1,065	137 (12.9%)		
3	207	26 (12.6%)		
≥4	76	13 (17.1%)		
**Single/double IUI**				
Single	2,352	267 (11.4%)	25.73	**<0.001**
Double	663	125 (18.9%)		
**Type of infertility**				
Primary	1,836	234 (12.7%)	0.273	0.601
Secondary	1,179	158 (13.4%)		
**Female age (years)**				
<30	1,042	143 (13.7%)	5.395	0.068
30–39	1,890	245 (13.0%)		
≥40	83	4 (4.8%)		
**Male age (years)**				
<30	649	88 (13.6%)	4.899	0.086
30–39	2,073	278 (13.4%)		
≥40	293	26 (8.9%)		
**Causes of infertility**				
Endometriosis after pelvic plasticity	49	12 (24.5%)	17.146	**0.046**
Immune infertility	141	29 (20.6%)		
Cervical	84	14 (16.7%)		
Ovulatory dysfunction	60	8 (13.3%)		
Male^*^/^**^	835	106 (12.7%)		
Unexplained infertility^*^/^**^	1,242	156 (12.6%)		
Others^*^/^**^	118	14 (11.9%)		
Both^*^/^**^	369	43 (11.7%)		
Multifactorial^*^/^**^	72	7 (9.7%)		
Endometriosis^*^/^**^	45	3 (6.7%)		

* *Means significantly different (P < 0.05) with the group of endometriosis after plasticity*.

** *Means significantly different (P < 0.05) with the group of immune infertility*.

*P values that below 0.05 are bold*.

Regarding the couples' ages, neither the male age factor nor the female age factor significantly affected the AIH pregnancy rate, but the female age factor was close to borderline significance (*P* = 0.068), and the pregnancy rates of the female age <30 years and 30–39 years groups were almost 3 times higher than that of the female age ≥40 years group (13.7% and 13.0 vs. 4.8%).

Among the infertility causes, the pregnancy rates of the endometriosis after plasticity group (24.5%) and the immune infertility group (20.6%) were significantly higher than those of the male factor group (12.7%), the unexplained group (12.6%), the other causes group (11.9%), the both male and female factor group (11.7%) and the endometriosis group (6.7%), with all *P* < 0.05.

### Association Between Sperm Parameters and AIH Pregnancy Rate

The Mann-Whitney-test was performed to compare the sperm concentration, viability, morphology, and motility parameters between the pregnant and non-pregnant groups (Table 2). Statistically significant differences were only found in VSL (14.31 vs. 13.75 μm/s, *P* = 0.048) and VCL (47.93 vs. 46.56 μm/s, *P* = 0.038).

**Table 2:** Comparison of sperm parameters between pregnant and non-pregnant groups.

	**Pregnant** **(*n*** = **392)**	**Non-pregnant** **(*n*** = **2,623)**	***p*-value**
Concentration (× 106/ml)	70.86 ± 53.92	71.28 ± 55.02	0.89
Motility (%)	93.58 ± 7.70	93.44 ± 7.98	0.894
TPMSC (× 106/ml)	32.88 ± 26.29	33.18 ± 26.90	0.923
VAP (μm/s)	24.73 ± 5.77	24.01 ± 5.90	0.08
VSL (μm/s)	14.31 ± 3.75	13.75 ± 3.61	**0.048**
VCL (μm/s)	47.93 ± 10.29	46.56 ± 10.78	**0.038**
ALH (μm/s)	6.60 ± 0.79	6.50 ± 0.81	0.072
BCF (Hz)	7.23 ± 0.69	7.24 ± 0.76	0.546
STR (VSL/VAP)	73 ± 7.82	72.14 ± 8.79	0.085
LIN (VSL/VCL)	47.74 ± 5.08	47.26 ± 5.77	0.155
Sperm head area (μm^2^)	5.59 ± 0.58	5.58 ± 0.58	0.848
SDI	1.24 ± 0.11	1.25 ± 0.11	0.43
TZI	1.3 ± 0.1	1.31 ± 0.1	0.67
H (%)	94.97 ± 4.43	95.26 ± 4.02	0.538
M (%)	16.71 ± 5.26	16.87 ± 5.08	0.421
P (%)	5.2 ± 3.14	5.55 ± 3.37	0.097
C (%)	7.13 ± 4.78	7.13 ± 4.93	0.784
Normal forms (%)	4.94 ± 4.37	4.56 ± 3.87	0.311
Abnormal forms (%)	95.05 ± 4.37	95.44 ± 3.87	0.255
Sperm survival rate (%)	82.75 ± 11.08	81.26 ± 12.79	0.057

*TPMSC, total progressive motile sperm count; VCL, curvilinear velocity; VSL, straight-line velocity; VAP, average pathway velocity; LIN, the linearity of movement; STR, straightness; BCF, beat cross frequency; ALH, the amplitude of lateral head displacement; SDI, sperm deformity index; TZI, teratozoospermia index; H, sperm headpiece deformity; M, sperm middle piece deformity; P, sperm principal piece deformity; C, sperm cytoplasm deformity. P values that below 0.05 are bold*.

### Multivariate Analysis of the Influencing Factors for AIH Pregnancy

**Figure 1:**
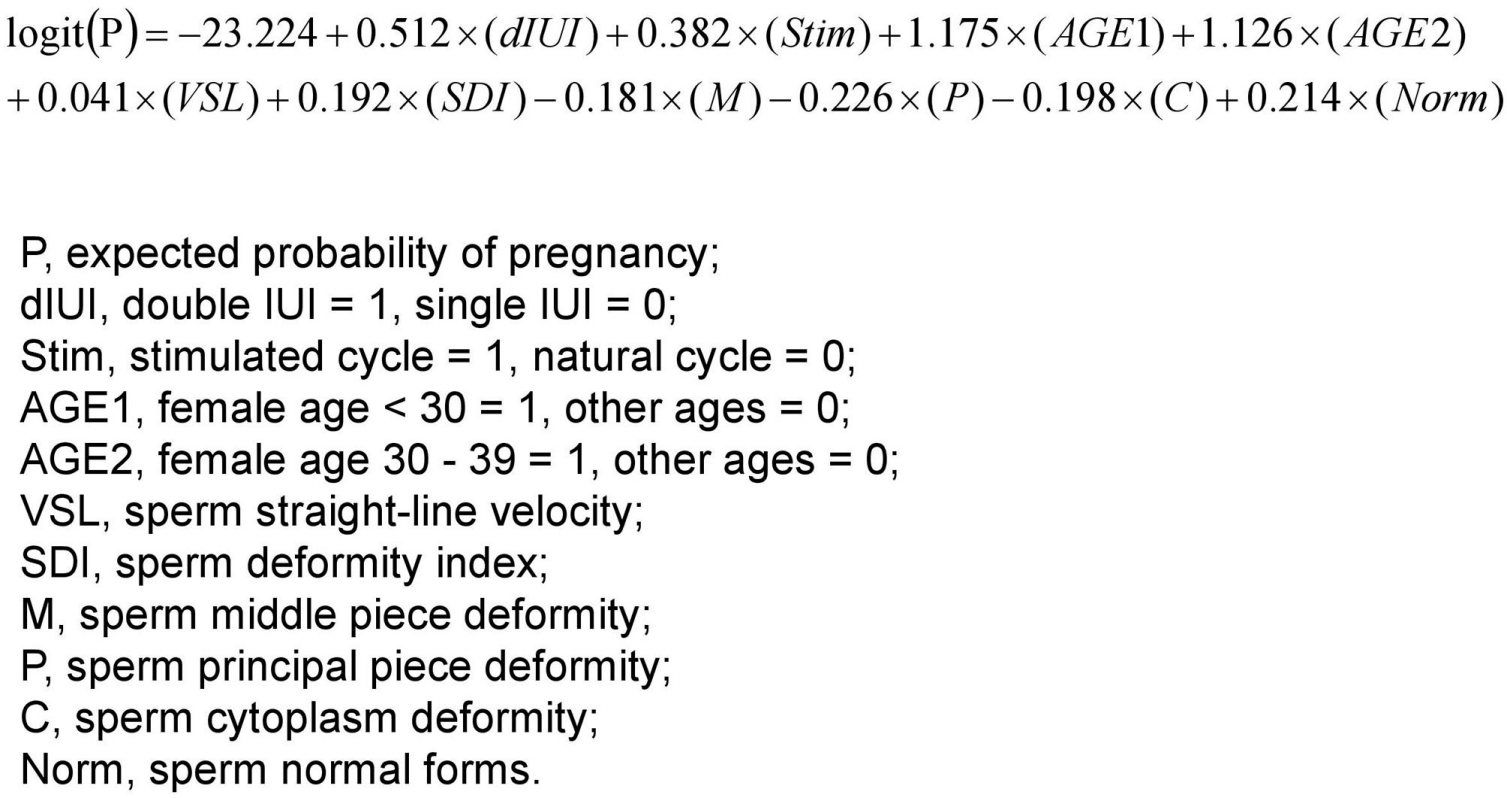
A multivariate logistic regression equation for predicting successful pregnancy following AIH. The 10 parameters were selected and weighted for predicting the probability of pregnancy *via* the multivariate binary logistic regression analysis performed by backward stepwise elimination. Thus, for each AIH case, the 10 parameters can be put into the equation to calculate the expected probability of pregnancy, which holds the potential to serve as a predictor for the outcome of the AIH treatment.

Parameters that might have an impact on AIH pregnancy outcomes, including cycle treatment options, single/double IUI, female age, male age, infertility causes, and sperm parameters (concentration, motility, TPMSC, VAP, VSL, VCL, ALH, BCF, STR, LIN, Sperm head area, SDI, TZI, H, M, P, C, normal form rate, abnormal form rate, survival rate), were entered into the logistic regression equation as independent variables, and pregnancy outcome as the dependent variable. Multivariate binary logistic regression analysis was performed by backward stepwise elimination to select factors that significantly impacted the AIH pregnancy outcome. As a result, there were 10 major influencing factors selected: stimulation treatment (OR = 1.466, 95% CI, 1.106–1.942, *P* = 0.008), double IUI (OR = 1.669, 95% CI, 1.309–2.129, *P* < 0.001), female age <30 years (OR = 3.238, 95% CI, 1.16–9.036, *P* = 0.025), female age 30–39 years (OR = 3.084, 95% CI, 1.113–8.544, *P* = 0.03), sperm VSL (OR = 1.042, 95% CI, 1.012–1.073, *P* = 0.006), SDI (OR = 1.211, 95% CI, 1.043–1.407, *P* = 0.012), sperm middle piece deformity (OR = 0.834, 95% CI, 0.717–0.97, *P* = 0.019), sperm principal piece deformity (OR = 0.798, 95% CI, 0.685–0.93, *P* = 0.004), sperm cytoplasm deformity (OR = 0.82, 95% CI, 0.703–0.956, *P* = 0.011) and sperm normal form rate (OR = 1.238, 95% CI, 1.062–1.444, *P* = 0.006) (Table 3). Based on the results, a multivariate logistic regression equation incorporating the above influencing factors was created for predicting AIH pregnancy outcome (Figure 1).

**Table 3:** Multivariate logistic regression analysis of influencing factors for AIH pregnancy.

	**Reference**	**β**	**Odd ratio (95% CI)**	***p*-value**
**Single/double IUI**				
Double IUI	Single IUI	0.512	1.669 (1.309–2.129)	**<0.001**
**Cycle treatment options**				
Stimulated cycle	Natural cycle	0.382	1.466 (1.106–1.942)	**0.008**
**Female age**				
<30	≥40	1.175	3.238 (1.16–9.036)	**0.025**
30–39	≥40	1.126	3.084 (1.113–8.544)	**0.03**
VSL		0.041	1.042 (1.012–1.073)	**0.006**
SDI		0.192	1.211 (1.043–1.407)	**0.012**
M		−0.181	0.834 (0.717–0.97)	**0.019**
P		−0.226	0.798 (0.685–0.93)	**0.004**
C		−0.198	0.82 (0.703–0.956)	**0.011**
Normal forms		0.214	1.238 (1.062–1.444)	**0.006**
Constant		−23.224	-	**0.002**

*β, regression coefficient; CI, confidence interval; VSL, straight-line velocity; SDI, sperm deformity index; M, sperm middle piece deformity; P, sperm principal piece deformity; C, sperm cytoplasm deformity. P values that below 0.05 are bold*.

The significantly influencing factors identified by multivariate logistic regression analysis were largely different from those identified by chi-square test or Mann-Whitney test, indicating that the prognostic values of many factors were actually affected by confounding factors.

### Assessment of Clinically Acceptable Predictors for AIH Pregnancy Outcome

**Figure 2:**
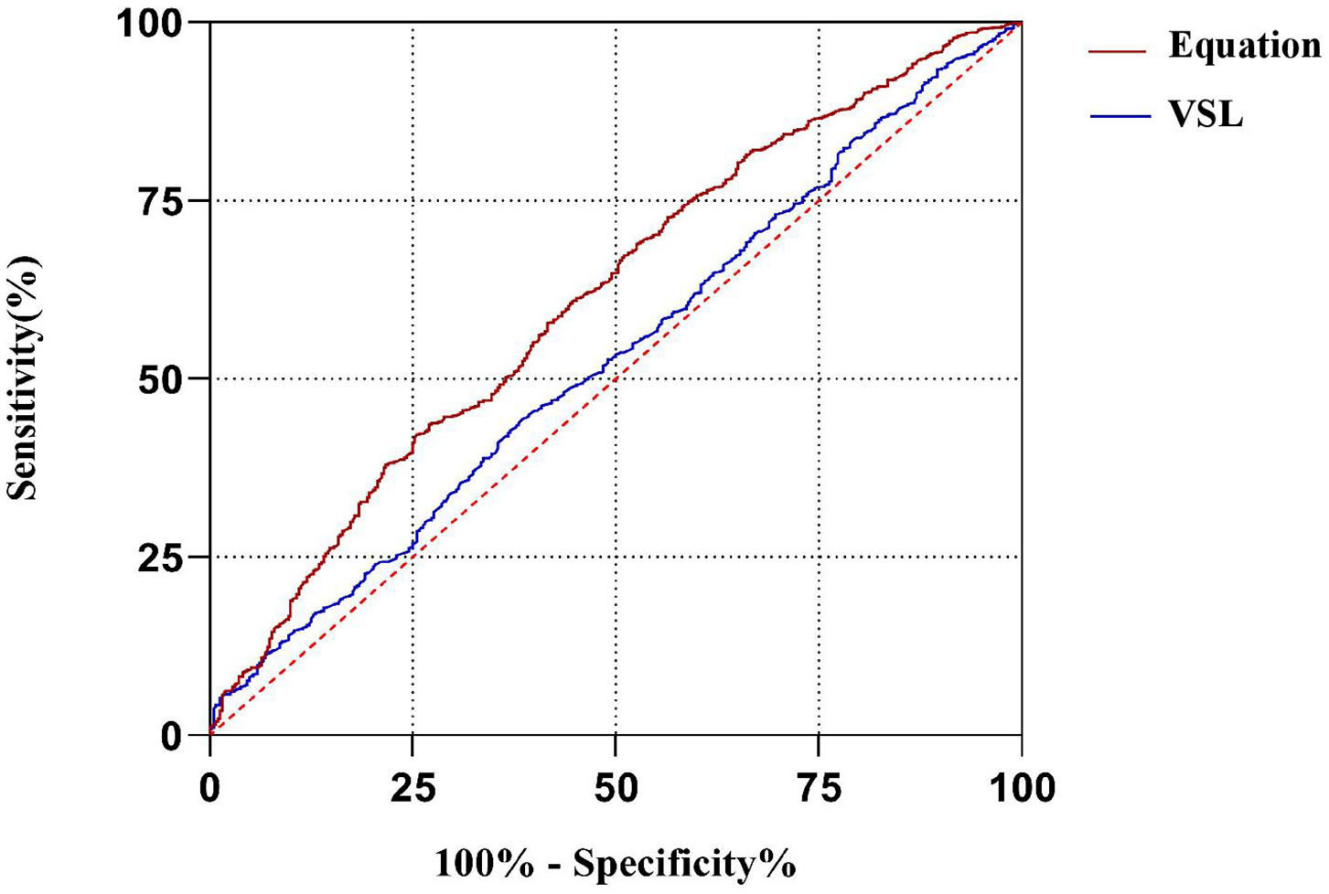
ROC curves of the equation and the predicting factor VSL. The area under curve (AUC) of the equation is much larger than that of VSL, indicating that the equation has better capacity of prognostic classification.

The multivariate logistic regression equation was applied to the study population to calculate the expected probability of pregnancy for each subject. This expected probability has the potential to serve as an integrated index for prognostic classification of AIH. The prognostic classification abilities for AIH pregnancy outcome of the independent influencing factors and the expected probability calculated by the equation were further assessed by ROC curves. The results showed that only the sperm VSL factor and the equation had significant values to serve as the prognostic classifier for AIH pregnancy (Table 4). And the clinically acceptable cut-off values were 13.22 μm/s for VSL and 11.34% for the logistic regression equation (Table 5). The statistical significance of the equation was dramatically higher than that of the sperm VSL factor (*P* < 0.001 vs. *P* < 0.048). In addition, the area under the curve (AUC) of the equation was also larger than that of the sperm VSL factor (Figure 2). Therefore, the equation had greater prognostic power than single predictors.

**Table 4:** ROC curve analysis of the influencing factors for AIH pregnancy.

	**AUC**	**95% CI**	***p*-value**
VSL	0.531 ± 0.015	0.501–0.561	**0.048**
SDI	0.488 ± 0.016	0.457–0.518	0.431
M	0.487 ± 0.016	0.456–0.519	0.421
P	0.474 ± 0.015	0.444–0.505	0.099
C	0.504 ± 0.016	0.474–0.535	0.784
Normal forms	0.516 ± 0.016	0.485-0.547	0.318
Equation	0.613 ± 0.015	0.583–0.643	**<0.001**

*ROC, receiver operating characteristic; AUC, the area under the curve; CI, confidence interval; VSL, straight-line velocity; SDI, sperm deformity index; M, sperm middle piece deformity; P, sperm principal piece deformity; C, sperm cytoplasm deformity. P values that below 0.05 are bold*.

**Table 5:** ROC curve analysis of the clinically acceptable cut-off values.

	**AUC**	**Cut-off**	**Sensitivity**	**Specificity**
VSL	0.531	13.22	61.5	44.4
Equation	0.613	11.34	74.5	42.2

*ROC, receiver operating characteristic; AUC, the area under the curve; VSL, straight-line velocity*.

## Discussion

Infertility can be caused by various factors. Due to the lack of knowledge of the etiological understanding, many infertility cases could only be circumvented by assisted reproductive technology (ART). In order to achieve successful fertility treatment, preparation of male or female gametes and the suitable physiological conditions in partners are sometimes critical. However, since fertilization is affected by multiple factors, to be able to diagnose the prerequisites of male and female contributions will improve the success rate. In the current study, we have analyzed a total of 3,015 tried AIH cycles and combined data to generate a mathematical model in order to facilitate the future treatments of patients with better predictable outcomes.

In our study, 392 out of 3,015 AIH cycles were successful, resulting in a 13% pregnancy rate, which is comparable to those of other reports (4, 18, 19). The impact of single/double IUI for achieving a pregnancy remains controversial. Arab-Zozani et al. reported that there was no evidence to support the use of double IUI in clinical practice (10). Polyzos et al. showed that double IUI offers no clear benefit in the overall clinical pregnancy rate in couples with unexplained infertility (20). On the contrary, Cantineau et al. described that the double IUI pregnancy rate was higher than the single IUI (21), and Dong et al. showed that the AID clinical pregnancy rate was significantly higher by double IUI than by single IUI AID (8). Our results supported that the pregnancy rate of double IUI was significantly higher than that of single IUI (18.9 vs. 11.4%) (Table 1). And the logistic regression analysis showed that the odds ratio of double IUI vs. single IUI was 1.669 (Table 3).

Similarly, whether ovulation stimulation is needed for IUI is also in a debate. Ye et al. described that ovulation induction did not result in a higher pregnancy rate, except for women over 35 years old (1). On the other hand, Li et al. have shown that the low-dose human menopausal gonadotrophin-mediated ovulation induction improved clinical pregnancy rates compared to natural cycles (9). Wan et al. found that, among women undergoing natural cycle IUI with donor sperm, hCG-triggered ovulation for timing insemination offers beneficial impacts on both clinical pregnancy rates and live birth rates (22). Monraisin et al. indicated that the use of GnRH antagonists has a positive effect on the delivery rate, especially in the multi follicular stimulations that are required when women are older than 27 years (11). Besides, in an IUI program for unexplained or mild male-factor infertility, Huang et al. believed that ovarian stimulation with letrozole may significantly increase live birth rates while controlling multiple pregnancy rates (23). Similarly, our results show that the pregnancy rate of ovulation stimulated cycles is significantly higher than that of natural cycles (14.4 vs. 9.0%) (Table 1). And logistic regression analysis showed that the odds ratio of stimulated cycles vs. natural cycles is 1.466 (Table 3).

Female age is another important influencing factor for the IUI pregnancy rate. Ashrafi et al. considered age 40 as a crucial threshold for a successful pregnancy (24); while others consider age 35 to be decisive (25, 26). And Vargas-Tominaga et al. suggested age 38 to be determinant, with a clinical pregnancy rate of 9.4% in women ?38 years compared to 5.6% in women ≥38 years (4). Albeit the literature shows different boundaries of female age, they all agreed that a woman's age affects the pregnancy rate following IUI. Similarly, our logistic regression analysis found that the female age was <30 years old and 30–39 years old the odds of pregnancy were 3.238 and 3.084 times of those in the age group ≥40 years old. Therefore, our results show that female age is an important factor affecting the success of IUI pregnancy, and the pregnancy rate decreases significantly after the age of ≥40 years (Tables 1, 3). So, this fact should be clearly emphasized in counseling of the 40-year-old that may opt for IUI.

At present, the impact of sperm parameters on the IUI pregnancy rate is still controversial. Omelet et al. pointed out that the sperm parameters most frequently examined concerning pregnancy rates included: (i) number of motile spermatozoa inseminated; (ii) sperm morphology using strict criteria; (iii) total motile sperm count in the native sperm sample; and (iv) total motility in the native sperm sample (27). Regarding sperm motility parameters, in earlier studies, it was found that VSL, VCL, ALH, and LIN are all related to fertility (16, 28, 29). Meanwhile, Youn et al. found no significant differences in these parameters between the pregnant and non-pregnant groups (17). In our study, we found borderline significant differences in VSL (*P* = 0.048) and VCL (*P* = 0.038) between the two groups (Table 2). After excluding confounding factors by logistic regression analysis, only VSL (OR = 1.04; *P* = 0.006) had a significant impact on the AIH pregnancy outcome (Table 3).

A large and growing body of literature has studied the influence of sperm morphology on the success rate of IUI. It is worth noting that some studies advise couples with ≤ 4% normal sperm morphology to go for IVF or ICSI instead of IUI (12, 30). On the contrary, there are other studies showing that sperm morphology has either no or low predictive value for pregnancy outcomes following IUI (14, 15, 31). Erdem et al. pointed out that the predictive value of morphological assessment in unexplained infertility is not reliable, but in male subfertility, the percentage of normal sperm morphology after washing is higher than 4.5%, which increases the live birth rate (32). Lemmens et al. also stated that sperm morphology ≤4% is more important in couples with male infertility factors (33). In our analysis of these morphology parameters between the pregnant group and the non-pregnant group, there was no significant difference between the two groups. However, after excluding the confounding effect by logistic regression analysis, SDI and sperm normal form rate significantly influenced the AIH pregnancy outcome, though ROC curve analysis showed that these parameters had no predictive value for prognostic classification. Therefore, we believe that sperm morphology has a certain impact on IUI pregnancy, but its predictive value needs to be further verified by a large number of studies, and the inclusion criteria of study subjects need to be strictly controlled and combined with the analysis of subgroups of various etiologies.

Some studies have reported the combination of several parameters for the prediction of successful pregnancy following IUI. For instance, Youn et al. found that sperm RAPID 30.1%, motility 51.4%, and concentration 111 × 10^6^/ml before sperm preparation could serve as good criteria for predicting the IUI pregnancy outcome of couples with unexplained infertility (17). In our study, we have developed a multivariate logistic regression equation incorporating both clinical characteristics and sperm parameters for prognostic classification of AIH pregnancy outcome. The reason for choose the logistic regression model is that it could remove the confounding factors, which refer to third-party factors that have correlations with both the exposed factor and the outcome, but are not elements in the cause-effect chain between the exposed factor and the outcome. Thus, if not excluded, they might falsely augment the causal effect when studying the correlation between the exposure and the outcome. After removing the confounding factors, the significant influencing factors identified by the logistic regression analysis were quite different from those identified by chi-square test or Mann-Whitney-test, implicating that single predictors could produce appreciable false-positive and false-negative results due to confounding effects. ROC curve analysis showed that the statistical significance and AUC of the multivariate logistic regression equation were much better than those of other independent influencing factors. Hence, the prognostic power of the equation is better than those of the single parameters.

## Conclusion

In summary, our study showed that the type of ovulation cycle, single/double IUI, female age, and sperm VSL, SDI, and normal form rate have significant impacts on the pregnancy rate of AIH in China. Furthermore, the multivariate logistic regression model incorporating the above influencing factors exhibited a greater power in predicting successful pregnancy than individual factors.

## Data Availability Statement

The raw data supporting the conclusions of this article will be made available by the authors, without undue reservation.

## Ethics Statement

The studies involving human participants were reviewed and approved by Third Affiliated Hospital of Guangzhou Medical University. Written informed consent for participation was not required for this study in accordance with the national legislation and the institutional requirements.

## Author Contributions

YL and DZ conceived and designed the study. HZ, JY, YZ, MZ, QL, and XS collected the samples, performed the assays, and acquired the data. SW, XX, YL, and DZ analyzed the data. SW, YL, and DZ wrote the manuscript. All authors read and approved the final manuscript.

## Conflict of Interest

The authors declare that the research was conducted in the absence of any commercial or financial relationships that could be construed as a potential conflict of interest.
